# Evaluation of Optic Nerve Head Parameters and Electro-Physiology Among Breast Cancer Patients on Tamoxifen

**DOI:** 10.7759/cureus.21042

**Published:** 2022-01-09

**Authors:** Qi Zhe Ngoo, Wan Hazabbah Wan Hitam, Chai Lee Tan, Venkata Murali Krishna Bhavaraju

**Affiliations:** 1 Ophthalmology, Universiti Sains Malaysia School of Medical Sciences, Kota Bharu, MYS; 2 Oncology, Universiti Sains Malaysia School of Medical Sciences, Kota Bharu, MYS

**Keywords:** pattern visual evoked potential, heidelberg retinal tomograph iii, optic nerve head parameters, toxic optic neuropathy, tamoxifen ocular toxicity

## Abstract

Introduction

To evaluate if early tamoxifen toxicity can be detected by comparing pre-and post-treatment optic nerve head parameters and visual function using Heidelberg Retinal Tomograph III (HRT III) and Pattern Visual Evoked Potential (Pattern VEP).

Method

This is a prospective study involving 76 eyes of 38 breast cancer patients treated with tamoxifen in Hospital Universiti Sains Malaysia, Kelantan, Malaysia. These patients were examined by a single doctor and the investigations were done by a single technician. The visual acuity, optic nerve function, visual field, optic nerve head parameters on HRT III and Pattern VEP were assessed. The examination was performed before and three months after treatment initiation.

Results

There was no tamoxifen ocular toxicity found three months post-treatment with tamoxifen. There was no change in visual acuity and optic nerve function post-treatment initiation. There were no statistically significant changes found in optic nerve head parameters on HRT III and P 100 peak latency and amplitude on Pattern VEP.

Conclusion

Ocular toxicity is a recognized complication of tamoxifen treatment. Tamoxifen optic neuropathy is a potentially irreversible, visually disabling complication. Tamoxifen ocular toxicity was not found three months after tamoxifen treatment initiation among estrogen receptor (ER)-positive breast cancer patients. No early changes in optic nerve head parameters and P 100 peak latency and amplitude changes were found after three months of treatment. A longer duration of monitoring with HRT III and Pattern VEP may be needed to adequately observe for early, subclinical changes in optic nerve head parameters and visual function among tamoxifen users.

## Introduction

Approximately two-thirds of breast cancer patients are positive for estrogen receptors (ER) and/or progesterone receptors (PR) at the time of diagnosis. Tamoxifen is a selective estrogen receptor modulator used in ER-positive breast cancer [1]. Tamoxifen acts by binding to ER and interfering with the binding of estrogen to ER, thus affecting tumor growth [2]. Tamoxifen has been reported to reduce recurrence and death rates after surgery for operable breast cancer, reduce contralateral breast cancer, and reduce the risk of invasive breast cancer by 49% in high-risk women [3-5]. The current standard treatment dosage is 20 mg per day for five consecutive years. However, recent trials showed that a longer duration of treatment of up to 10 years may reduce the recurrence of disease and mortality.

Ocular toxicity was first described in female breast cancer patients who received very high doses of tamoxifen, 120 to 130 mg per day [6]. Subsequent reports found that ocular toxicity can also occur with standard low-dose tamoxifen treatment [7-8]. The incidence of tamoxifen-related ocular side effects ranges from 6.3 to 12% [9-10]. The spectrum of tamoxifen-related ocular toxicity includes keratopathy, cataract [11], crystalline retinopathy, macular edema [12], pseduocystic foveal cavitation [13,14], and optic neuritis [15]. Resolution of tamoxifen-induced keratopathy has been reported upon cessation of treatment [16]. Retinopathy and optic neuropathy caused by tamoxifen, if detected early, are likewise reversible [17].

The duration for tamoxifen therapy required to induce ocular toxicity varies. Ocular toxicity due to tamoxifen may occur as early as a few weeks after initiating the medication, or even years later. Gianni et al. reported that tamoxifen ocular toxicity occurs after a median of 10 months of treatment [18]. Impairment of visual function has been described in patients treated with a standard tamoxifen dosage [19-20].

Optic neuritis is a less common but potentially irreversible visual morbidity from tamoxifen ocular toxicity. It affects bilaterally and can occur as early as three weeks after starting tamoxifen treatment. Detection of tamoxifen-induced toxic effects on the optic nerve before the occurrence of symptoms is of great value to prevent extensive optic nerve damage and furthermore allow the complete recovery of normal function.

Anatomical changes in the fundus or optic nerve due to tamoxifen toxicity were previously documented by conventional ophthalmoscopy examination or photography. Confocal laser scanning ophthalmoscopy has revealed significantly smaller optic cups in visually asymptomatic patients on short-term tamoxifen treatment [21]. Several case reports showed prolonged latency in patients with tamoxifen-induced optic neuritis. Visual evoked potential (VEP) is recognized as a sensitive measure of optic nerve pathologies. It is more sensitive for the diagnosis of resolved optic neuritis than visual acuity, contrast sensitivity, Goldmann perimetry, or magnetic resonance imaging [22,23].

As early ocular toxicity may not manifest clinically, assessment of optic nerve head and visual function using various tests may be useful to detect subclinical changes in patients on tamoxifen. We thus aim to detect subclinical changes of optic nerve head on Heidelberg Retinal Tomograph III (HRT III) and Pattern Visual Evoked Potential (Pattern VEP) before manifestations of tamoxifen-induced ocular toxicity are present.

## Materials and methods

This is a cross-sectional prospective study involving 76 eyes of 38 patients diagnosed with breast cancer who completed primary treatment and were under follow-up at the oncology clinic of Hospital Universiti Sains Malaysia (HUSM). The study was conducted in the eye clinic of HUSM. The study was approved by the Human Research Ethics Committee (HREC) Universiti Sains Malaysia with approval number 1403118. This study was conducted following the tenets of the declaration of Helsinki. All participating patients have signed informed consent.

Patients who were planned for treatment with 20 mg daily tamoxifen and who fulfilled the inclusion and exclusion criteria were invited to participate prior to treatment initiation. Informed written consent was obtained from each patient. A total of 38 patients were included in the study after exclusion of: (1) underlying ocular disease, (2) preexisting corneal opacity or presence of dense ocular opacity, (3) abnormal HRT III or Pattern VEP on initial evaluation, (4) systemic illness including diabetes mellitus, hypertension or renal impairment, (5) patients with a history of taking neurotoxic medications, (6) mentally challenged patients, (7) developmental brain disease or with brain metastasis.

Visual acuity measurement was measured monocular using a Snellen chart for distance (Reichert, NY, USA) at 6 meters. A comprehensive ocular examination, including pupillary examination, color vision, visual field, slit-lamp biomicroscopy of the anterior and posterior segment, and intraocular pressure measurement by Goldmann applanation tonometer was performed to rule out preexisting ocular conditions which would have precluded participation in the study.

HRT III imaging for both eyes was performed by a single trained technologist. A good quality image was considered when the following criteria were fulfilled: (1) the optic disc image appeared centrally, (2) minimal eye movement detected during image capture, (3) absence of artifact, and (4) topography standard deviation less than 50 micrometers. Only images with good and acceptable overall quality scores were used for analysis. A mean topographic image was obtained from three to four scans (15 degrees field of view). Manual drawing of the optic disc margin was performed by the same technologist and was compared to Moorfield Regression Analysis.

Pattern VEP was performed based on the standard International Society for Clinical Electrophysiology of Vision (ISCEV) Pattern VEP protocol 2009. Pattern VEP was tested monocularly with appropriate refractive correction. The stimulus used in this study was elicited by checkerboard stimuli with a large 1º check. Value of peak latency P100, N75, and N135, and amplitude of N75- P100 and P100 - N135 were measured.

Ocular examination, optic nerve head parameters measurement with HRT III, and Pattern VEP were repeated after three months of tamoxifen treatment. A data collection sheet was used to document all relevant parameters. Statistical analyses were performed using IBM SPSS version 22.0 (IBM Corp., Armonk, NY). Paired t-test was performed to determine the changes of optic nerve head parameters and Pattern VEP measurements pre-treatment and after three months.

## Results

A total of 76 eyes of 38 female patients were recruited in this study. Their mean age was 43.3±5.5 years. Four (11%) patients were postmenopausal. The majority of patients were Malay (35, 92.1%), followed by Chinese (2, 5.3%) and Siamese (1, 2.6%). All patients were non-smokers. All patients had a visual acuity of 6/6 in one eye and 6/9 or better visual acuity in the other eye. No corneal opacities, cataracts, refractive opacities within the retina, macular edema, or optic neuritis were found after three months of treatment.

The means ± SD of Heidelberg Retinal Tomography III parameters for both eyes are shown in Tables 1-2. We do not find statistically significant changes in optic nerve head parameters after three months of treatment.

**Table 1:** Comparison of optic nerve head parameters for the right eye before and after three months of treatment with tamoxifen among breast cancer patients. (n=38) * Paired t-test was applied. SD = standard deviation, MD = mean difference, df = degree of freedom, RNFL = retinal nerve fiber layer.

Optic nerve head parameters	Mean (SD)		Mean Difference (95% CI)	t-statistics (df)	p-value*
	Pre	3 months			
Disc area (mm^2^)	2.16 (0.46)	2.17 (0.46)	0.01 (-0.01, 0.02)	0.86 (37)	0.394
Cup area (mm^2^)	0.49 (0.26)	0.49 (0.26)	-0.00 (-0.01, 0.00)	-1.53 (37)	0.135
Rim area (mm^2^)	1.59 (0.48)	1.59 (0.48)	0.00 (-0.00, 0.01)	0.20 (37)	0.845
Cup volume (mm^3^)	0.11 (0.09)	0.11 (0.09)	0.00 (-0.00,0.00)	0.40 (37)	0.691
Rim Volume (mm^3^)	0.48 (0.15)	0.47 (0.14)	-0.00 (-0.01, 0.00)	-1.41 (37)	0.168
Cup/Disc Area Ratio	0.23 (0.11)	0.22 (0.10)	-0.00 (-0.01, 0.00)	-1.97 (37)	0.057
Mean cup depth (mm)	0.21 (0.08)	0.22 (0.08)	0.00 (-0.00, 0.01)	1.02 (37)	0.311
Maximum cup depth (mm)	0.62 (0.17)	0.62 (0.17)	-0.00 (-0.01, 0.01)	-0.05 (37)	0.960
Cup shape measure	-0.22 (0.07)	-0.22 (0.06)	-0.00 (-0.01, 0.01)	-0.17 (37)	0.867
Height variation contour (mm)	0.42 (0.08)	0.42 (0.08)	0.00 (-0.01, 0.01)	0.07 (37)	0.946
Mean RNFL thickness (mm)	0.28 (0.05)	0.28 (0.06)	0.00 (-0.01, 0.01)	0.24 (37)	0.812
RNFL cross-sectional area (mm^2^)	1.49 (0.28)	1.49 (0.28)	0.00 (-0.01, 0.01)	0.19 (37)	0.847

**Table 2:** Comparison of optic nerve head parameters for the left eye before and after three months of treatment with tamoxifen among breast cancer patients. (n=38) * Paired t-test was applied. SD = standard deviation, MD = mean difference, df = degree of freedom, RNFL = retinal nerve fiber layer.

Optic nerve head parameters	Mean (SD)		Mean Difference (95% CI)	t-statistics (df)	p-value*
	Pre	3 months			
Disc area (mm^2^)	2.19 (0.44)	2.19 (0.44)	0.00 (-0.01, 0.02)	1.64 (37)	0.109
Cup area (mm^2^)	0.49 (0.30)	0.49 (0.30)	-0.00 (-0.01, 0.00)	-1.18 (37)	0.246
Rim area (mm^2^)	1.66 (0.45)	1.66 (0.45)	-0.00 (-0.00, 0.01)	-1.74 (37)	0.091
Cup volume (mm^3^)	0.10 (0.11)	0.10 (0.10)	0.00 (-0.00, 0.00)	0.24 (37)	0.809
Rim volume (mm^3^)	0.47 (0.15)	0.47 (0.15)	0.00 (-0.01, 0.01)	0.28 (37)	0.780
Cup/Disc Area Ratio	0.22 (0.12)	0.22 (0.11)	-0.00 (-0.01, 0.00)	-1.03 (37)	0.310
Mean cup depth (mm)	0.20 (0.08)	0.20 (0.08)	-0.00 (-0.01, 0.00)	-0.47 (37)	0.639
Maximum cup depth (mm)	0.58 (0.18)	0.57 (0.18)	-0.00 (-0.01, 0.00)	-1.02 (37)	0.313
Cup shape measure	-0.21 (0.07)	-0.22 (0.07)	-0.00 (-0.01, 0.00)	-1.42 (37)	0.164
Height variation contour (mm)	0.43 (0.07)	0.44 (0.07)	0.01 (-0.00, 0.01)	1.99 (37)	0.054
Mean RNFL thickness (mm)	0.28 (0.05)	0.28 (0.06)	0.00 (-0.00, 0.01)	1.59 (37)	0.121
RNFL cross-sectional area (mm^2^)	1.46 (0.29)	1.46 (0.28)	0.00 (-0.01, 0.01)	0.47 (37)	0.644

Table 3 shows the Pattern VEP mean ± SD of latency and amplitude of both eyes. No statistically significant reduction of P 100 latency and amplitude were observed among patients after three months.

**Table 3:** Comparison of Pattern VEP latency and amplitude for the right eye before and after three months of treatment with tamoxifen among breast cancer patients. (n=38) * Paired t-test was applied. SD = standard deviation, MD = mean difference, df = degree of freedom, Pattern VEP = Pattern visual evoked potential

Variables	Mean (SD)		Mean Difference (95% CI)	t-statistics (df)	p-value*
	Pre	3 months			
N 75 1 degree latency (ms)	83.71 (4.56)	84.47 (4.60)	0.76 (-0.13, 1.65)	1.74 (37)	0.090
P 100 1 degree latency (ms)	111.34 (3.14)	111.71 (3.06)	0.37 (-0.19, 0.93)	1.34 (37)	0.190
N 135 1 degree latency (ms)	140.68 (8.53)	142.79 (7.46)	2.11 (-0.57, 4.78)	1.60 (37)	0.119
N 75 – P 100 amplitude (μV)	12.23 (4.43)	12.12 (4.18)	-0.12 (-0.83, 0.59)	-0.33 (37)	0.742
P 100 – N 135 amplitude (μV)	13.36 (5.01)	13.28 (4.91)	-0.09 (-0.54, 0.37)	-0.39 (37)	0.696

## Discussion

Breast cancer and tamoxifen

Breast cancer is the leading cause of cancer-related death among women worldwide [24]. Based on the National Cancer Registry 2007-2011, breast cancer is the most common malignancy among women in Malaysia. Although the prevalence is lower in Asian countries than in developed countries, it is still an important women’s health issue, as it is slowly becoming the commonest female malignancy in Asia [25]. Tamoxifen is an important drug in the management of ER-positive breast cancer [26].

Tamoxifen was introduced in 1967. It is a non-steroidal estrogen antagonist which plays a role in combating and preventing breast cancer in women [27]. The National Surgical Adjuvant Breast and Bowel Project (NSABP) trial found tamoxifen may play a role as a chemopreventive agent in women with a higher risk for the disease. Tamoxifen is generally taken at a dose of 20 mg or 40 mg daily for five years, depending on the cancer stage. However, the global Adjuvant Tamoxifen: Longer Against Shorter (ATLAS) trial has recently shown the continuation of tamoxifen treatment to 10 years, can further reduce the recurrence and mortality of breast cancer patients [28].

There are notably some potential side effects for this drug including ocular effects. Toxic optic neuropathy is one of the most dreaded complications. Although it is rare, the outcome of optic neuropathy is profound and distressing as there have been cases where the condition was irreversible. Estrogen may have a neuroprotective role; thus, the use of tamoxifen may disrupt the astrocytes in the glial cell of the optic cup that contains estrogen receptors at the optic nerve head. This may result in swelling of the optic nerve head, thus reducing the cup volume.

Optic nerve head parameters

Studies have shown that during follow-up, patients on tamoxifen have reduced cup volume. This could be possible due to the postulation mentioned above. However, there is another postulation is that the cup size changes due to hormonal alterations [29].

In our study, we noted that there was no statistically significant difference in the changes of the optic disc cup size pre and three months post tamoxifen initiation. However, we observed optic cup area, cup disc area ratio, maximum cup depth, and cup shape measure showed a slight decrease three months post-treatment. We postulate that these findings may indicate possible very early, evolving changes of the optic nerve head parameters.

Electrophysiological tests

Pattern VEP is an electrophysiological test that can assist in the diagnosis of ocular conditions and is useful in the diagnosis of subclinical optic neuropathy. In cases of established tamoxifen optic neuropathy, prolonged P 100 latency and a decrease in amplitude in Pattern VEP have been reported [30]. However, based on the PubMed search, there is no prospective study on Pattern VEP conducted among tamoxifen users to evaluate tamoxifen-related optic neuropathy subclinical detection to determine the duration and dosage.

There was an average of increased latency of the P100 values in our study; however, these were not statistically significant. The literature showed that most cases of toxic optic neuropathy are dependent on dose and duration. Our study was conducted for three months with the recommended dosage of 20 mg daily, we postulate early subclinical optic neuropathy may still be evolving. Prolonging our study for a longer duration with repetition of serial electrophysiological tests at regular intervals may allow us to monitor the progression of these changes.

Limitations

One limitation of our study was its short duration. Toxic optic neuropathies in many conditions are generally dose and duration-dependent. The higher the dose, the higher the likelihood of developing optic neuropathy. Tamoxifen optic neuropathy was reported to occur as early as three weeks to seven months of treatment. However, based on one study, the median time for tamoxifen ocular toxicity was approximately 10 months post-treatment. The small sample size was another limitation of our study. A larger sample size may reflect more precise possible early subclinical changes of optic nerve parameters and optic nerve. We suggest patients on tamoxifen not only be followed up for the duration of their treatment, but also after cessation of therapy. This may give a more accurate evaluation of the drug’s effects on the eyes.

## Conclusions

Tamoxifen ocular toxicity can affect the cornea, lens, retina, macula, and optic nerve. Tamoxifen optic neuropathy is an important complication due to its potentially devastating effect on vision. No tamoxifen-induced ocular toxicity was found among breast cancer patients three months after standard dose treatment initiation. No statistically significant changes seen in optic nerve head parameters on HRT III and P 100 latency and amplitude from Pattern VEP after three months on tamoxifen. Ocular toxicity from tamoxifen may occur at a variable interval after initiation of treatment, therefore baseline and serial monitoring with HRT III and serial electrophysiology test may provide an insight of early, subclinical progression of tamoxifen-related optic neuropathy.
